# Impact of alcohol consumption and body mass index on mortality from nonneoplastic liver diseases, upper aerodigestive tract cancers, and alcohol use disorders in Korean older middle-aged men: Prospective cohort study

**DOI:** 10.1097/MD.0000000000004876

**Published:** 2016-09-30

**Authors:** Sang-Wook Yi, Jae-Seok Hong, Jee-Jeon Yi, Heechoul Ohrr

**Affiliations:** a Department of Preventive Medicine and Public Health, Catholic Kwandong University College of Medicine; b Institute for Clinical and Translational Research, Catholic Kwandong University, Gangneung; c Department of Healthcare Management, Cheongju University College of Health Sciences, Cheongju; d Institute for Occupational and Environmental Health, Catholic Kwandong University, Gangneung; e Institute for Health Promotion, Graduate School of Public Health, Yonsei University, Seoul; f Department of Preventive Medicine, Yonsei University College of Medicine, Seoul, Republic of Korea.

**Keywords:** alcohol drinking, alcohol-related disorders, body mass index, esophagus cancer, liver diseases, mortality

## Abstract

Alcohol use is a leading risk factor for the global disease burden including liver diseases. However, the combined effect of alcohol use and body mass index (BMI) on alcohol-related diseases has seldom been examined. We examined whether alcohol consumption and BMI could act together to increase mortality from nonneoplastic liver diseases, upper aero-digestive tract (UADT) cancers, and alcohol use disorders (AUD) in middle-aged Korean men.

107,735 men (mean age, 58.8 years) participated in a postal survey in 2004 and were followed until 2010, by linkage to national death records. Hazard ratios (HRs) of cause-specific death were calculated after adjustment for confounders.

Each 5-drink (approximately 45 g alcohol) higher weekly alcohol consumption was associated with increased mortality, by approximately 70% for nonneoplastic liver disease mortality (HR = 1.70, *P* < 0.001), approximately 60% for UADT cancer mortality (HR = 1.64, *P* < 0.001), and approximately 70% for AUD mortality (HR = 1.71, *P* < 0.001). Generally, BMI was inversely associated with these alcohol-related diseases (HR per each 5 kg/m^2^ higher BMI = 0.18-0.46, *P* < 0.001 for each cause), while, in participants with BMI ≥25 kg/m^2^, each 5 kg/m^2^ higher BMI was also associated with an elevated mortality from nonneoplastic liver diseases of approximately 150% (HR = 2.52, *P* = 0.001). Men with BMI < 21 kg/m^2^ and weekly alcohol consumption ≥14 drinks showed markedly higher mortality from nonneoplastic liver diseases (HR = 5.7), alcoholic liver diseases (HR = 9.3), UADT cancers (HR = 10.5), and esophageal cancer (HR = 15.5), compared to men drinking less than 1 drink/wk with BMI ≥25 kg/m^2^. The combined effect of low BMI and high weekly alcohol consumption was 2.25- to 3.29-fold greater than the additive effect of each factor for these alcohol-related diseases (*P* < 0.05 for each cause).

Alcohol consumption and low BMI were related to deaths from nonneoplastic liver diseases, UADT cancers, and AUD, with evidence of a supra-additive combined effect of both factors. High BMI was also related to deaths from nonneoplastic liver diseases. Men with a low BMI (<23 kg/m^2^) are suggested to be prone to the harmful effects of alcohol.

## Abstract 2

Supplemental Digital Content is available in the text

## 1 Introduction

Globally, 3.3 million deaths are attributable to alcohol use every year.^[1]^ Alcohol use is considered the third leading risk factor contributing to the global disease burden in men.^[2]^ Liver diseases, which are a leading cause of death and have increased in recent years in the United Kingdom and United States,^[3]^ are closely linked with alcohol consumption, as are alcohol use disorders (AUD) and upper aerodigestive tract (UADT) cancers, such as oral cavity, larynx, and esophagus cancers. Previous research has suggested that low body mass index (BMI) is associated with liver diseases, UADT cancers, and AUD,^[4–10]^ while liver diseases are also associated with high BMI.^[8–10]^ The joint effect of alcohol consumption and BMI on these alcohol-related diseases, however, has been infrequently examined and is not well understood.^[4,9–12]^ Additionally, associations between BMI and mortality from these alcohol-related diseases per se have not been clearly established.^[9–11]^ A better understanding of the impact of alcohol consumption and body weight on these alcohol-related diseases may help physicians and health professionals in treating their patients and establishing strategies to reduce the burden of these diseases. We aim to evaluate whether alcohol consumption and BMI interact to increase the risk of death from these alcohol-related diseases, based on a study of middle-aged Korean men. The independent effect of each factor was also examined.

## 2 Methods

### 2.1 Study population

Among 187,897 men initially included in the Korean Veterans Health Study,^[13,14]^ 164,208 living men were identified in June 2004 after the exclusion of 23,689 individuals who were deceased or had emigrated. A postal survey was sent out on July 27, 2004, to which 117,609 men (71.6%) replied. Those with missing information on BMI (n = 3693) or alcohol consumption (n = 5743), or whose residential status was uncertain after the initial survey (n = 438) were excluded from the present study. Ultimately, 107,735 men were included in the analysis. This study was approved by the Institutional Review Board of Kwandong University. Since all able-bodied men are required to serve in the military in Korea, this study cohort is not necessarily an occupational cohort. The participants had lower overall mortality (sex–age–calendar year standardized mortality ratio [SMR] = 0.79, 95% confidence interval [CI] = 0.77–0.81) than the general Korean population.

### 2.2 Ascertainment of causes of death

Follow-up on deaths and their underlying causes from August 1, 2004 to December 31, 2010 was confirmed by national death records through record linkage and was complete. The International Classification of Diseases 10th Revision (ICD-10) was used to define alcohol-related causes of death, which were classified into nonneoplastic liver diseases (K70-K76), alcoholic liver disease (K70), UADT cancers (C00–C15, C30–C32), esophagus cancer (C15), and AUD (F10; mental and behavioral disorders due to use of alcohol). Liver cancer was not included in this study due to the lack of a clear dose–response relationship with alcohol consumption.

### 2.3 Data collection and estimation of weekly alcohol consumption

Information on smoking, alcohol consumption, physical activity, height, weight, and income was collected from the survey. BMI (kg/m^2^) was calculated from the self-reported weight in kilograms divided by the square of the self-reported height in meters. More details about the survey can be obtained elsewhere.^[15]^ Participants with prevalent UADT cancers diagnosed from January 1, 1992 to July 31, 2004 were assessed through the National Cancer Incidence Database.^[14]^ Participants were recorded as having prevalent nonneoplastic liver diseases, viral hepatitis (B15–B19), or substance use disorders (F10–F19) if they visited a medical institution at least once for such a condition between January 1, 2000 and July 31, 2004.

Participants were asked to answer the questions “How often did you drink alcoholic beverages over this past year?” and “How many drinks of alcoholic beverages did you have on a typical drinking day?” The drinking frequency was reported as daily (estimate of drinking days per week, 7), 5–6 d/wk (5.5), 3–4 d/wk (3.5), 1–2 d/wk (1.5), 2–3 d/mo (0.58), 1 d/mo (0.23), 7–11 d/y (0.17), 4–6 d/y (0.1), 2–3 d/y (0.05), 1 d/y (0.02), none for a year (0), or never have drunk (0). Alcohol consumption (drinks) per drinking day was evaluated using an open question, with instructions that a bottle of *soju* (360 mL) contains 7 drinks, a can of beer (355 mL) 1.4 drinks, and so on. Weekly alcohol consumption (drinks) was estimated by multiplying the amount of alcohol consumption on a drinking day by the number of drinking days per week. *Soju* (a distilled alcoholic beverage native to Korea) generally contained 21% to 22% alcohol and beer 4% to 5% alcohol at the time of the survey in 2004. The amount of ethanol in a standard drink across all types of alcoholic beverages was approximately 9 g.

Respondents were classified into 5 groups according to weekly alcohol consumption defined by drinks per week (<1 [reference], 1–6, 7–13, 14–27, and ≥28) and into 3 groups (<1 [reference], 1–13, and ≥14). Drinking frequency and alcohol consumption per drinking day were also classified into 5 and 3 categories, respectively. These alcohol consumption-related variables were additionally analyzed as continuous variables.

### 2.4 Statistical analysis

In the main analysis, no adjustment for preexisting disease status was performed in order to minimize collider-stratification bias and to increase the generalizability of our findings.^[16,17]^ Men with prevalent diseases were excluded in a subgroup analysis.

BMI values were categorized into 7 groups (<18.5, 18.5–20.9, 21.0–22.9, 23.0–24.9, 25.0–27.4 [Reference], 27.5–29.9, ≥30 kg/m^2^) using cut-off points suggested by the World Health Organization,^[18]^ and into 3 groups (<21, 21–24.9, ≥25 kg/m^2^ [reference]).^[18]^ The reference BMI category was selected based on previous research in East Asian men that showed the lowest mortality at about 25 to 27 kg/m^2^.^[19,20]^

Cox proportional hazards models were used to calculate hazard ratios (HRs) after adjusting for the following covariates: age at enrollment (continuous variable), smoking (never, past, current smoker, or those with missing information [n = 873]), physical activity (yes, or no activity); monthly household income (<500,000, 500,000–990,000, 1,000,000–1,490,000, and ≥1,500,000 Korean won, and those with missing information [n = 4186]). In order to evaluate the combined effect of BMI and alcohol consumption, 9 combined groups of BMI and alcohol-related variables were constructed using 3 categories of both BMI and alcohol-related variables. The obese/overweight participants with the least alcohol consumption were used as the reference category. Subgroup analyses were done in which former drinkers (n = 7411; eTable 1), or men who had preexisting diseases related to each cause of death were excluded (eTable 2).

In order to assess the biological interaction (departure from additivity) between BMI (low BMI vs high BMI, with a low BMI defined as <23 kg/m^2^) and the alcohol-related variables (high vs low-intermediate weekly alcohol consumption, with high consumption being defined as ≥14 drinks per week), the Synergy Index was calculated using previously described methodologies.^[21]^ The Synergy Index represents how many times higher the combined effect is than the additive effect of 2 factors. If no biological interaction is present, the Synergy Index is equal to 1.

*P*-values were calculated using 2-sided tests. All statistical analyses were performed using SAS version 9.4 (SAS, Inc., Cary, NC).

## 3 Results

During 669,920 person-years of follow-up in men (mean age: 58.8 years at baseline), 338 men died from nonneoplastic liver diseases, 199 from UADT cancers, and 41 from AUD. The proportions of underweight and obesity were 2.5% and 1.2%, respectively (Table 1). The heavier drinkers tended to be somewhat younger, slightly more obese, and more likely to be current smokers, and had higher household incomes.

**Table 1:**
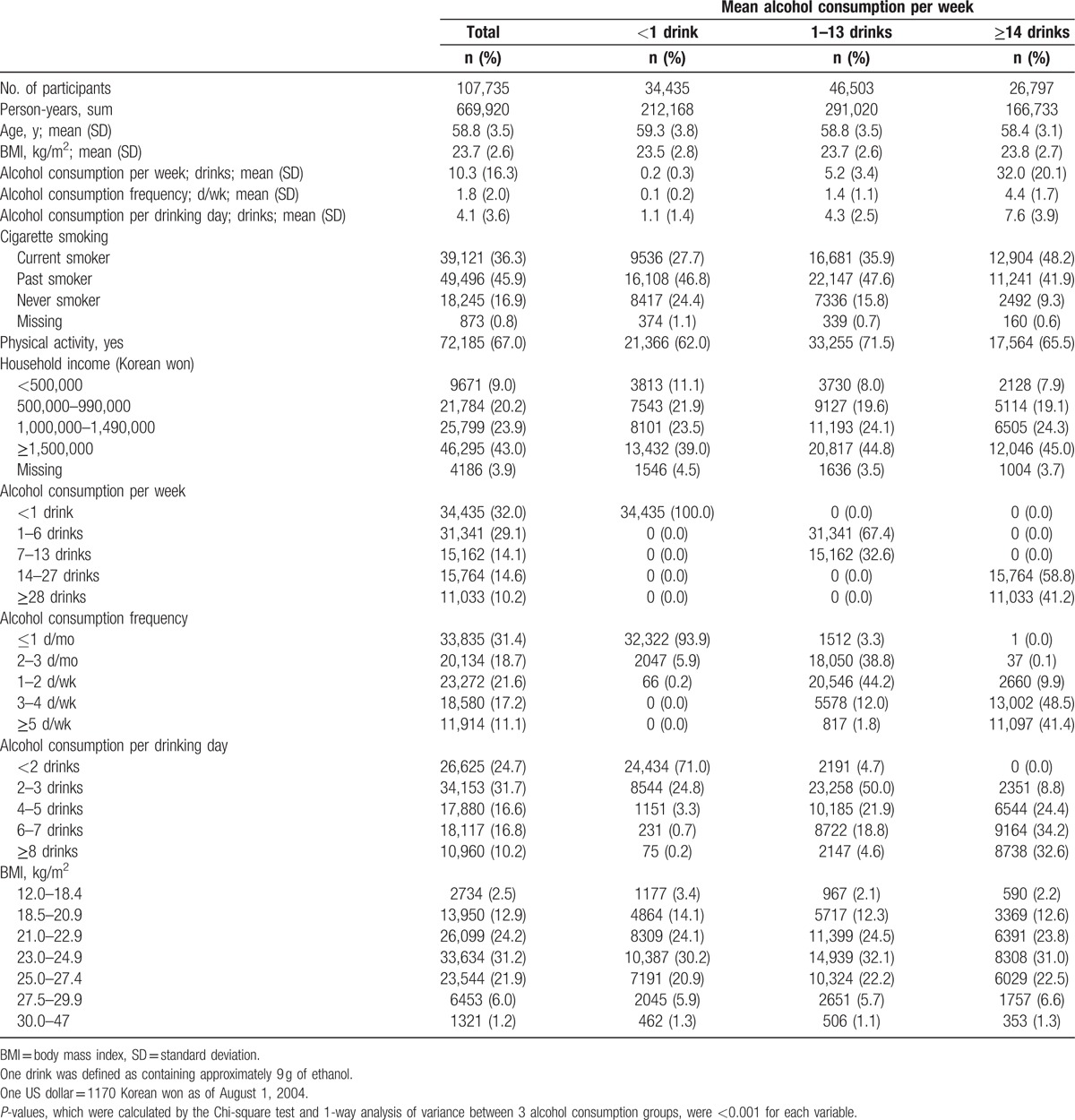
Characteristics of participants according to alcohol consumption.

Higher levels of weekly alcohol consumption were strongly associated with increased mortality from each disease (Table 2, eTables 3 and 4). Each 5-drink increase per week was associated with 60% to 70% higher risk of mortality from nonneoplastic liver diseases, UADT cancers, and AUD. When past drinkers or people with preexisting diseases relevant to the outcomes were excluded, the HRs associated with higher alcohol consumption strengthened, rather than weakened (eTables 1 and 2).

**Table 2:**
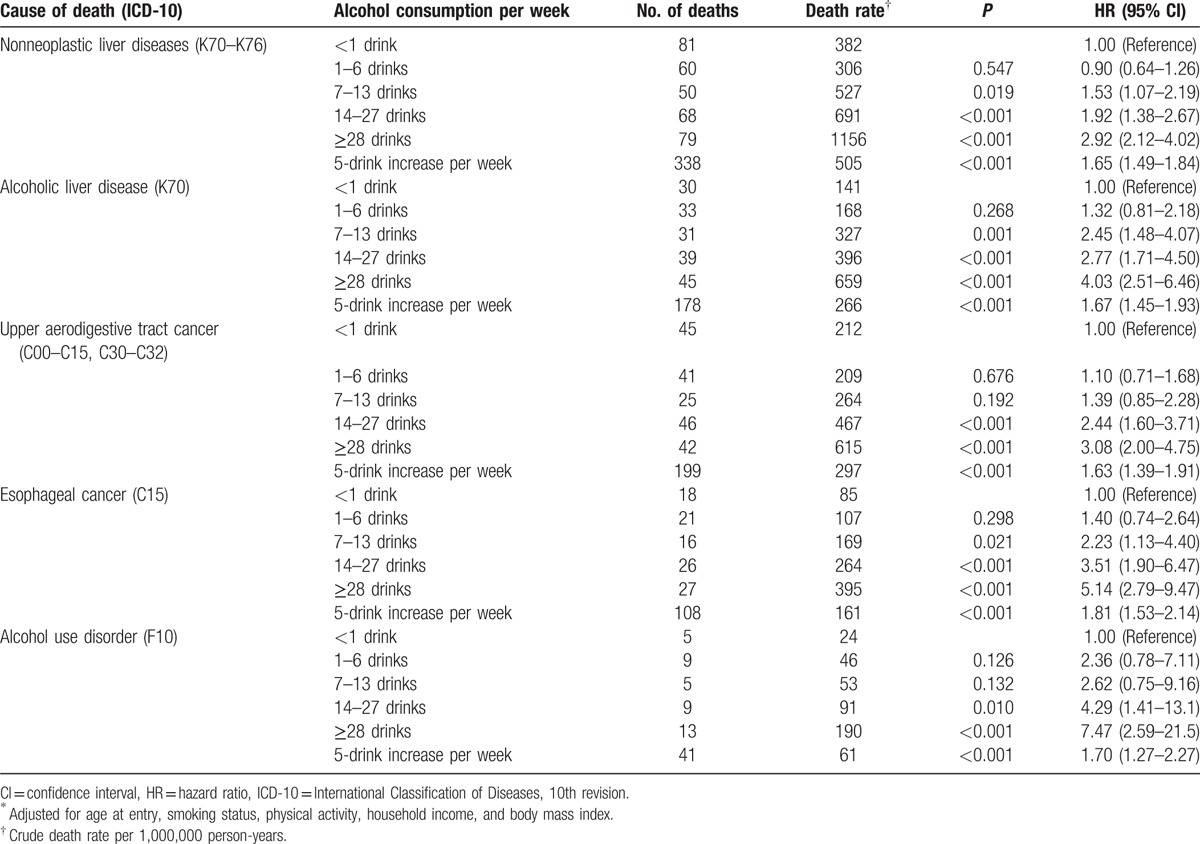
Death rates and adjusted^∗^ hazard ratios for cause-specific mortality by weekly alcohol consumption among Korean older middle-aged men.

**Figure 1:**
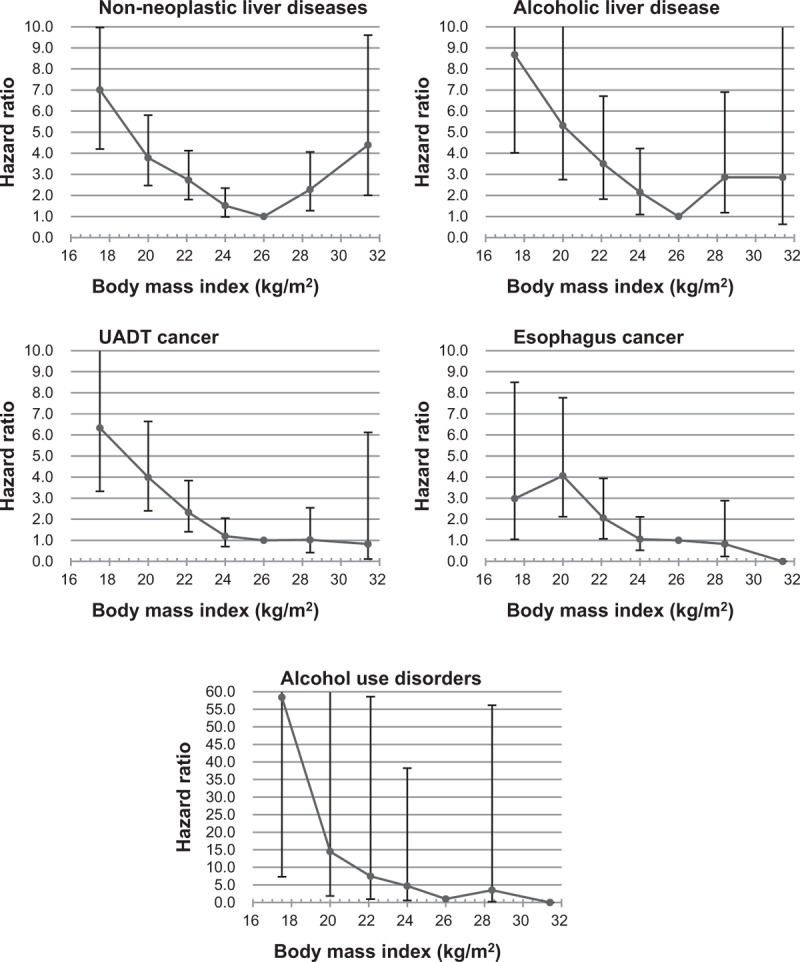
Hazard ratios of cause-specific mortality according to body mass index (BMI). Hazard ratios were calculated using Cox proportional hazard models. Seven BMI categories (<18.5, 18.5–20.9, 21–22.9, 23–24.9, 25–27.4 [reference], 27.5–27.9, and ≥30 kg/m^2^) were used. The mean value was used as a representative value of each BMI category. The analyses were adjusted for age, cigarette smoking, physical activity, household income, and weekly alcohol consumption (5 groups). For esophageal cancer and alcohol use disorders, no deaths were observed in the BMI ≥ 30 categories. UADT = upper aerodigestive tract.

BMI showed a U-curve association with nonneoplastic liver diseases (including alcoholic liver diseases), while BMI was inversely associated with mortality from other alcohol-related diseases (Fig. 1). Each 5 kg/m^2^ higher BMI was associated with lower mortality from nonneoplastic liver diseases (HR = 0.46, 95% CI = 0.37–0.56; eTable 5), alcoholic liver disease (HR = 0.40, 95% CI = 0.30–0.53), UADT cancers (HR = 0.32, 95% CI = 0.24–0.42), esophagus cancer (HR = 0.36, 95% CI = 0.25–0.52), and AUD (HR = 0.18, 95% CI = 0.10–0.33). When restricted to BMI ≥25 kg/m^2^, each 5 kg/m^2^ higher BMI was associated with higher mortality from nonneoplastic liver diseases (HR = 2.52, 95% CI = 1.49–4.26) and alcoholic liver disease (HR = 2.13, 95% CI = 0.80–5.66). When men with preexisting diseases relevant to the outcomes were excluded, the general inverse associations remained relatively unchanged, whereas higher mortality rates from nonneoplastic liver diseases, including alcoholic liver disease, showed weakened associations with high BMI (eFigure 1, eTable 6).

In the stratified analyses using 3 BMI groups, the highest alcohol consumption group showed a strong positive association with mortality from alcohol-related diseases among men with BMIs of 12 to 20.9 and 21 to 24.9 kg/m^2^, while the associations were generally weak in overweight/obese men (eTable 7).

**Figure 2:**
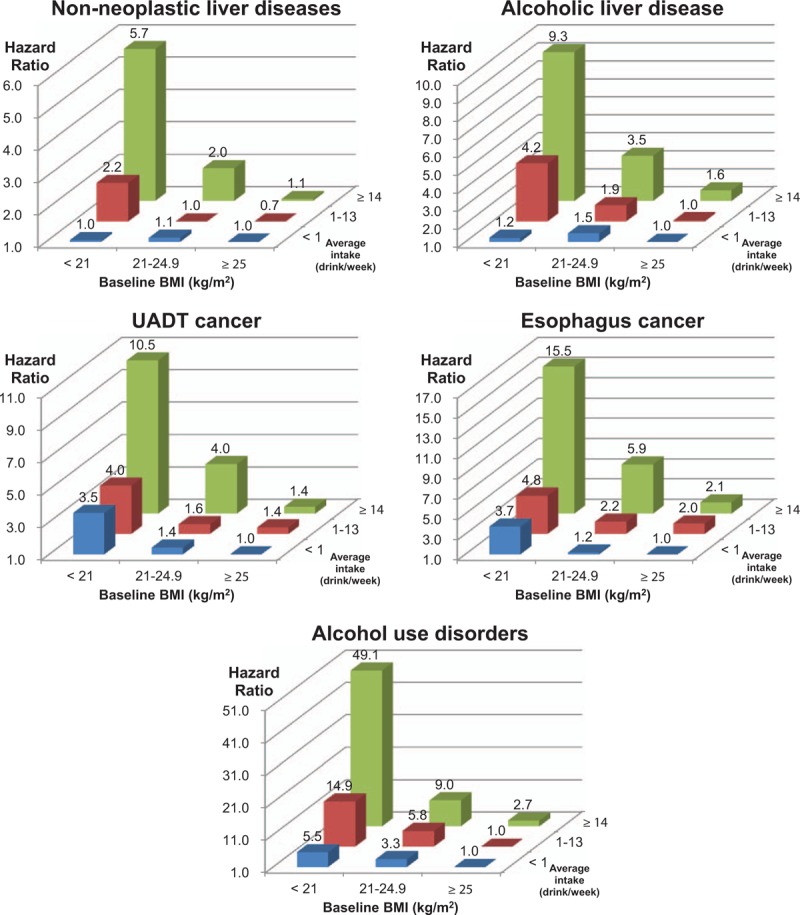
Hazard ratios of cause-specific mortality according to 9 groups combining body mass index (BMI) and weekly alcohol consumption. Hazard ratios were calculated using Cox proportional hazard models after adjusting for age, cigarette smoking, physical activity, and household income. Persons with a BMI ≥ 25 kg/m^2^ and weekly alcohol consumption of less than 1 drink were the reference group. One drink was defined as containing approximately 9 g of ethanol. For alcohol use disorders, persons with a BMI ≥25 kg/m^2^ and weekly alcohol consumption of 0 to 13 drinks (<1 or 1–13 drinks/wk) were the reference group, due to the absence of deaths in persons with a BMI ≥ 25 kg/m^2^ and an alcohol consumption of less than 1 drink/wk.

In the analyses using 9 groups combining BMI and alcohol-related variables, higher alcohol consumption was associated with a higher risk of death from alcohol-related diseases, as BMI decreased (Fig. 2, eFigures 2–4).

**Figure 3:**
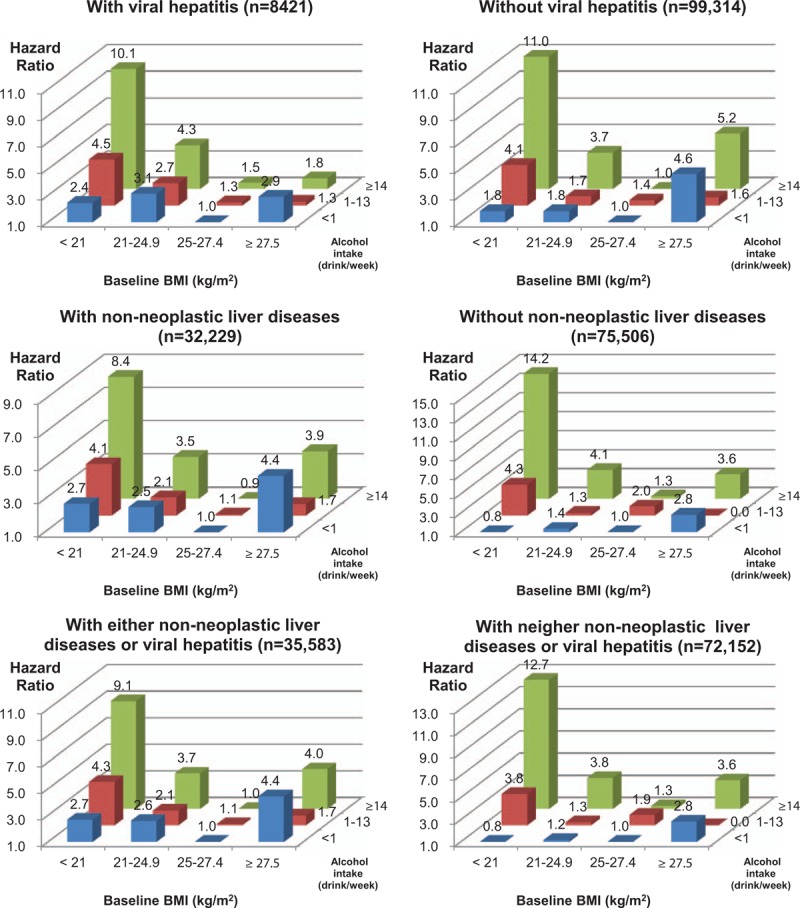
Hazard ratios of mortality from nonneoplastic liver diseases according to 12 groups combining body mass index (BMI) and weekly alcohol consumption in persons with or without viral hepatitis and nonneoplastic liver diseases at baseline. Hazard ratios were calculated using Cox proportional hazard models after adjusting for age, cigarette smoking, physical activity, and household income. Persons with a BMI 25 to 27.4 kg/m^2^ and weekly alcohol consumption of less than 1 drink were the reference group. One drink was defined as containing approximately 9 g of ethanol. Participants were recorded as having prevalent nonneoplastic liver diseases (K70–K76) or viral hepatitis (B15–B19) at baseline if they visited a medical institution at least once for a given condition between January 1, 2000 and July 31, 2004.

Meanwhile, in persons with low alcohol consumption (less than 1 drink per week), BMI < 21 was not associated with higher mortality from nonneoplastic liver diseases (including alcoholic liver disease) compared with BMI ≥25 (Fig. 2), or compared with BMI 25 to 27.4 in men without prevalent nonneoplastic liver diseases (Fig. 3).

**Figure 4:**
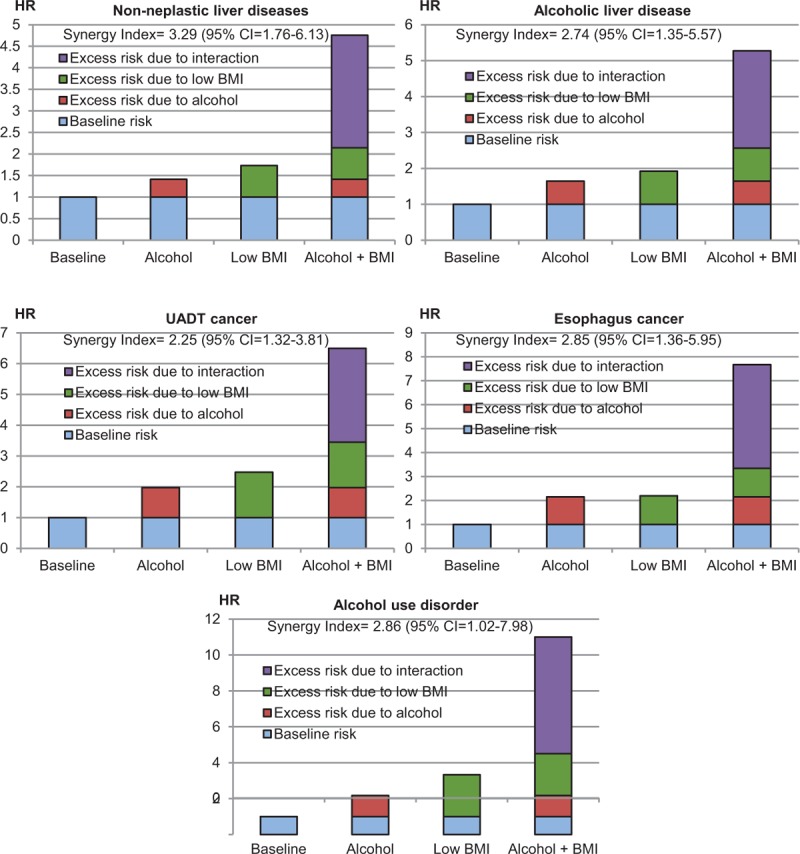
Hazard ratios (HRs) of the contributions of alcohol consumption and low body mass index (BMI) to cause-specific mortality. Alcohol consumption was classified into 2 groups (≥14 vs 0–13 drinks/wk), while BMI was classified into 2 groups (<23 vs ≥23 kg/m^2^). The HRs were calculated using Cox proportional hazard models after adjusting for age, cigarette smoking, physical activity, and household income. Persons with a BMI ≥ 23 kg/m^2^ and a weekly alcohol consumption of 0 to 13 drinks were the reference group. The *Synergy Index* represents how many times higher the combined effect (interaction) was than the additive effect of alcohol consumption and low BMI, with a value of 1 indicating no biological interaction. CI = confidence interval.

In the 4-group analysis used to evaluate the biological interaction between BMI and alcohol consumption, the Synergy Index indicated that the combined effect of low BMI and high weekly alcohol consumption on mortality from these alcohol-related diseases was 2.25- to 3.29-fold greater than the additive effect of each factor (Fig. 4; *P* < 0.05 for each cause).

## 4 Discussion

In this large prospective cohort study, alcohol intake displayed a strong linear association with mortality from nonneoplastic liver diseases, UADT cancers (including esophagus cancer), and AUD. Each 5-drink (approximately 45 g alcohol) increase in weekly alcohol consumption was associated with increased mortality, by approximately 60% to 70% for mortality from nonneoplastic liver diseases, UADT cancers, and AUD. Low BMI was generally associated with higher mortality from these alcohol-related diseases, while high BMI was also strongly associated with higher mortality from nonneoplastic liver diseases. Among participants with BMI ≥ 25 kg/m^2^, each 5 kg/m^2^ increment in BMI increased mortality from nonneoplastic liver diseases by approximately 150%. The combined effect of low BMI and high alcohol consumption was substantially greater than the additive effect of each factor analyzed independently.

### 4.1 Association between BMand alcohol-related diseases

Both low and high BMI were associated with increased mortality from liver diseases, in accordance with 2 large cohort studies.^[8,9]^ The magnitude of relative risk estimates seemed to be greater both for low and high BMIs in the present study than in previous research.^[8–10,12,22]^ Regarding UADT cancers, the observed inverse associations between BMI and UADT cancers (including esophagus cancer, mostly squamous cell carcinoma^[5]^) concur with the majority of, but not all,^[23]^ previous studies.^[4–6]^ The associations of body weight with alcohol consumption and AUD remain controversial.^[24]^ Associations between BMI and mortality from AUD have infrequently been evaluated. Our study found a strong inverse relationship.

### 4.2 Combined effects of BMand alcohol consumption on alcohol-related mortality

Two prospective studies have examined the combined effect of BMI and alcohol consumption on liver diseases.^[9,10]^ A study in UK men found a supra-additive interaction between high BMI and alcohol consumption. In the Million Women Study in the United Kingdom, obesity contributed independently to the mortality rate of liver cirrhosis, but not to the relative risk. When we further classified the overweight range into 25 to 27.4 and ≥27.5 kg/m^2^ to evaluate the combined effect of high BMI and alcohol consumption, we found no evidence of supra-additive interaction (eFigure 5). Regarding UADT cancers, several case–control studies have suggested that low BMIs may enhance the effects of alcohol consumption on the risk of oral cavity and oropharyngeal cancer^[4,25]^; however, prospective studies have not confirmed this joint effect.^[11]^ Few studies have examined the joint effect on esophagus cancer or AUD.

Our analysis clearly showed that low BMI and high alcohol consumption independently increased the mortality from alcohol-related diseases, with a supra-additive combined effect for each disease. However, low BMI may be more an effect modifier of alcohol consumption than an independent risk factor for nonneoplastic liver diseases, since, in persons with low weekly alcohol consumption (less than 1 drink), low BMI was not associated with higher mortality (Fig. 2), especially in persons without prevalent liver diseases (Fig. 3).

### 4.3 Potential mechanisms

Large cohort studies have found low BMIs (e.g., below 22.5) to be associated with higher liver diseases mortality.^[8,9]^ In a general Scottish population, BMI was inversely associated with hospitalization due to alcohol-related harm, including alcoholic liver disease and liver cirrhosis.^[22]^ Several case–control studies found a synergic effect of low BMI on the association between alcohol consumption and UADT cancers.^[4,25]^ These studies, however, provided no clear explanation. Increased alcohol-related mortality associated with low BMI, as well as a supra-additive combined effect of low BMI and higher alcohol consumption, may be partly explained by higher alcohol consumption relative to body weight in persons with lower BMI than with higher BMI. The overweight/obese men consumed the least amount relative to their body weight (0.14 drinks/wk/kg body weight in men with BMI ≥ 25 kg/m^2^; 0.18 drinks/wk/kg body weight in men with BMI < 21 kg/m^2^; *P* < 0.001) in the present study in accordance with previous findings that higher BMI was associated with lower blood alcohol concentrations.^[26]^ Therefore, the harmful effects of alcohol may be greater in persons with lower BMIs than with higher BMIs. For nonneoplastic liver diseases, sarcopenia (the decline of muscle mass and strength) may partially explain the observed associations. Sarcopenia may be associated with liver fibrosis,^[27]^ and it becomes more prevalent as BMI decreases.^[28]^ Thus, a potential synergic interaction between sarcopenia and alcohol consumption may increase the mortality rate in persons with a lower BMI. Regarding higher liver disease mortality in persons with BMI ≥ 27.5, mechanisms such as the crosstalk between dysfunctional and insulin-resistant adipocytes and the liver may promote the development of fatty liver disease, which may subsequently lead to liver cirrhosis, a fatal liver disease.^[29]^ Additionally, genetic traits, such as those linked to the alcohol dehydrogenase 1B (ADH1B) gene,^[30]^ which is related both to alcohol metabolism and BMI, might have played a role in the observed findings.

### 4.4 Implications

Our study clearly showed that, even if lower BMI per se is not a risk factor, the harmful effect of alcohol consumption on these alcohol-related diseases was substantially greater in persons with a lower BMI. Persons with low-normal weight (18.5–22.9 kg/m^2^), should be recognized as prone to the harmful effects of alcohol—if not for all alcohol-related diseases, then at least for nonneoplastic liver diseases, UADT cancers, and AUD. In obese persons, weight reduction may substantially lower the burden of nonneoplastic liver diseases.

### 4.5 Strengths and limitations

The prospective design of this study is a clear strength. Due to the large number of events, we were able to reliably evaluate the effect of alcohol consumption and BMI. Follow-up was complete, meaning that no bias was present due to loss to follow-up. Since preexisting diseases were identified using the nationwide database, bias related to self-reported information regarding preexisting conditions was minimized, while conditions important for assessing specific causes of mortality were controlled for in the analyses (e.g., viral hepatitis infection for nonneoplastic liver diseases). This study also has several limitations. BMI was calculated based on self-reported height and weight. Alcohol consumption was assessed using a self-reported questionnaire. However, self-reported measures of alcohol intake are known to be reasonably valid and reliable.^[31]^ BMI and alcohol consumption may have changed over the follow-up period. Since the Korean sample was slim overall, this study had a limited ability to detect associations in the obese range. Generalizability may be a limitation, since the participants were Koreans and had Vietnam War experience. Our study participants had a lower mortality than the general male population of Korea (SMR = 0.79), while Koreans have a life expectancy comparable to those of other Organization for Economic Co-operation and Development (OECD) populations. Since the associations of BMI with mortality may differ by ethnicity, region, sex, and age,^[19]^ some of our findings may need to be confirmed in other populations. However, the effects of alcohol consumption and BMI were generally consistent for several alcohol-related diseases over several alcohol consumption measures in various subgroup and sensitivity analyses. This aspect of our main findings may make them more generalizable to other populations.

## 5 Conclusions

This large prospective cohort study in Korean men found that higher alcohol consumption was strongly associated with increased mortality from nonneoplastic liver diseases, UADT cancers, and AUD. High BMI (≥27.5 kg/m^2^) was strongly associated with higher mortality from nonneoplastic liver diseases, while low BMI was strongly associated with higher mortality from nonneoplastic liver diseases, UADT cancers, and AUD. The harmful effects of alcohol consumption on mortality from these alcohol-related diseases were generally greater as BMI decreased. The combined effect of low BMI and high alcohol consumption was greater than the independent additive effects of these 2 factors. For nonneoplastic liver diseases, low BMI may be more of an effect modifier of alcohol consumption than a risk factor per se.

## Acknowledgment

The authors sincerely thank the staff of the Korean National Statistical Office for providing the mortality data used herein.

## Supplementary Material

Supplemental Digital Content

## Supplementary Material

Supplemental Digital Content
