# Supplementary material for: Impact of alcohol consumption and body mass index on mortality from nonneoplastic liver diseases, upper aerodigestive tract cancers, and alcohol use disorders in Korean older middle-aged men: Prospective cohort study
Source: Medicine (Baltimore). 2016 Sep 30;95(39):e4876. doi: 10.1097/MD.0000000000004876 (PMC5265912; doi:10.1097/MD.0000000000004876)
Supplement: Supplemental Digital Content [file medi-95-e4876-s001.doc]

0


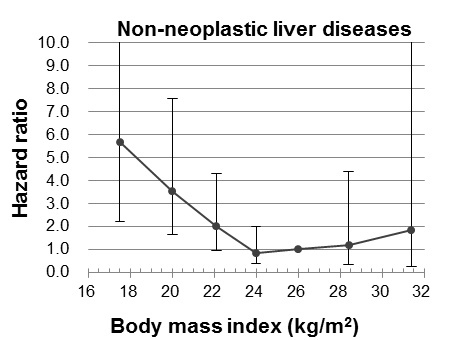

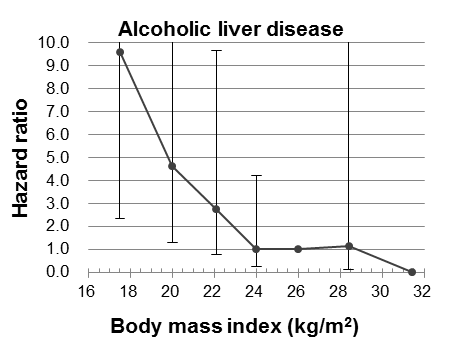

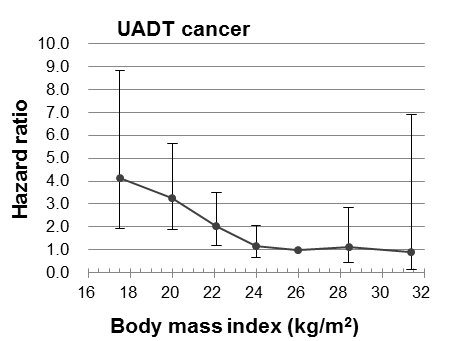

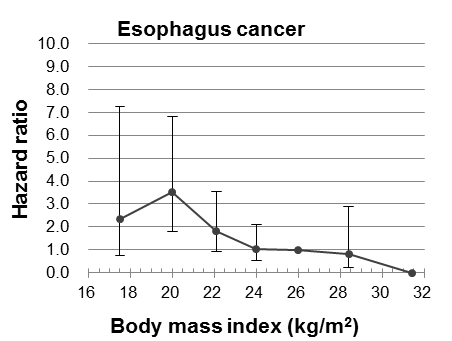

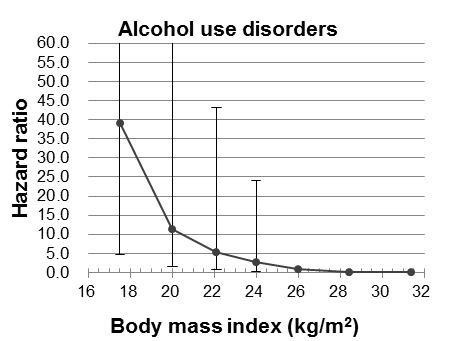


**eFigure 1. Hazard ratios of cause-specific mortality according to body mass index (BMI) after excluding pre-existing diseases relevant to the outcomes.** Hazard ratios were calculated using Cox proportional hazard models. Seven BMI categories (<18.5, 18.5–20.9, 21–22.9, 23–24.9, 25–27.4 [reference], 27.5–27.9, ≥30 kg/m^2^) were used. The mean value was used as a representative value of each BMI category. The analyses were adjusted for age, cigarette smoking, physical activity, household income, and weekly alcohol consumption (five groups). For some causes, no death was observed in the BMI ≥30 kg/m^2^ or 27.5–29.9 kg/m^2^ categories. UADT, upper aerodigestive tract.

0


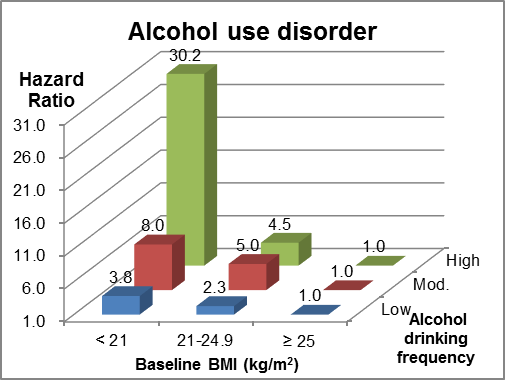

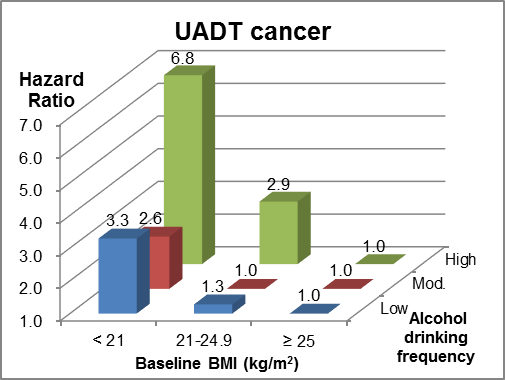

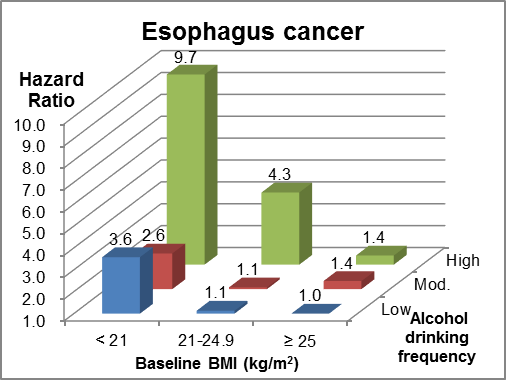

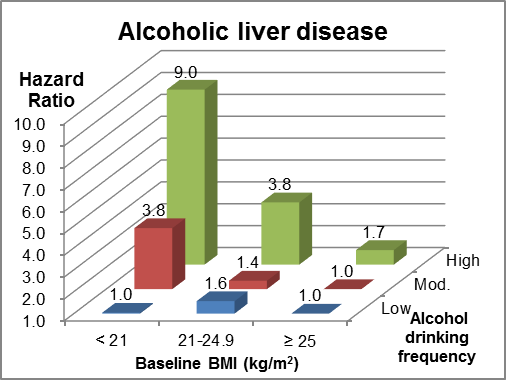

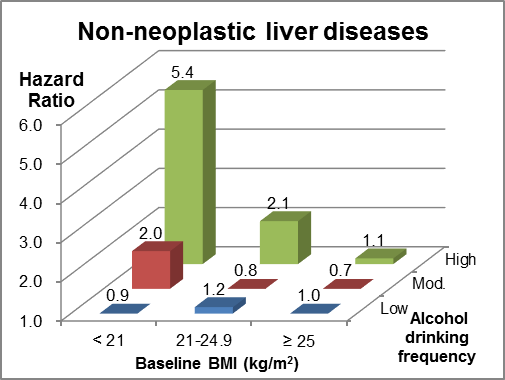


**eFigure 2. Hazard ratios of cause-specific mortality across nine groups combining body mass index (BMI) and alcohol intake frequency.** Alcohol intake frequency was classified into three categories (low, one or fewer drinking days per month; moderate, two days/month to two days/week; high, three to seven days/week). Hazard ratios were calculated using Cox proportional hazard models after adjusting for age, cigarette smoking, physical activity, and household income. Persons with a BMI ≥25 kg/m^2^ and a low frequency of alcohol consumption were the reference group. For alcohol use disorders, persons with a BMI ≥25 kg/m^2^ and all alcohol frequency groups combined were the reference group, due to the absence of deaths in persons with BMIs ≥25 kg/m^2^ and a low-to-moderate frequency of alcohol consumption. UADT, upper aerodigestive tract.

0


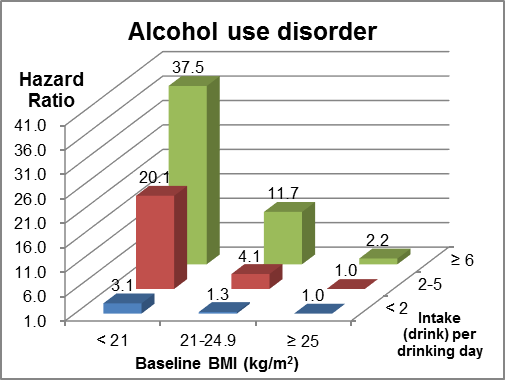

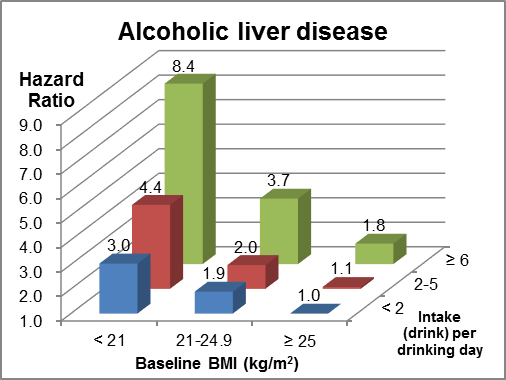

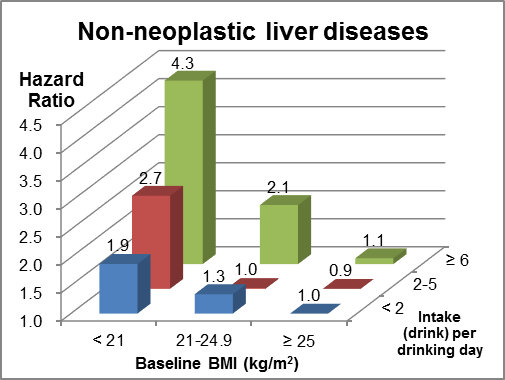

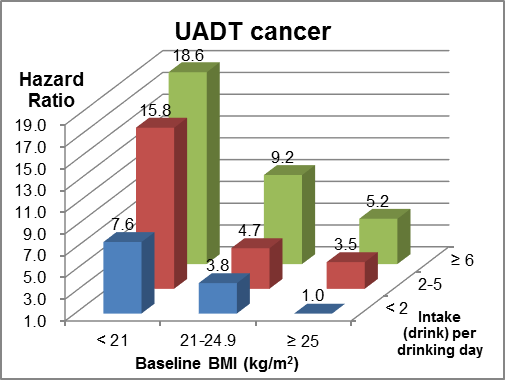

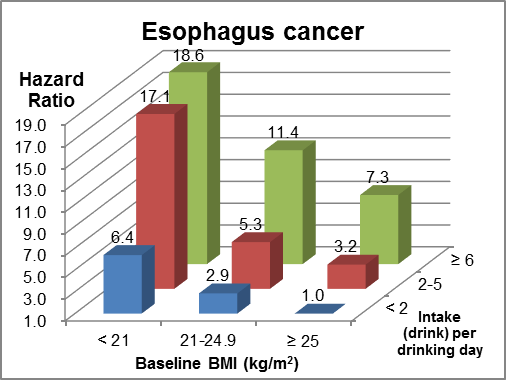


**eFigure 3. Hazard ratios of cause-specific mortality according to nine groups combining body mass index (BMI) and alcohol intake per drinking day.** Alcohol intake per drinking day was categorized into three groups (fewer than two, two to five, and six or more drinks/day). Hazard ratios were calculated using Cox proportional hazard models after adjusting for age, cigarette smoking, physical activity, and household income. Persons with a BMI ≥ 25 kg/m^2^ who consumed fewer than two drinks per drinking day were the reference group. One drink was defined as containing approximately 9 g ethanol. For alcohol use disorders, persons with a BMI ≥ 25kg/m^2^ who consumed less than six drinks per drinking day were the reference group, due to the absence of deaths in persons with a BMI ≥ 25 kg/m^2^ who consumed fewer than two drinks per drinking day. UADT, upper aerodigestive tract.

0


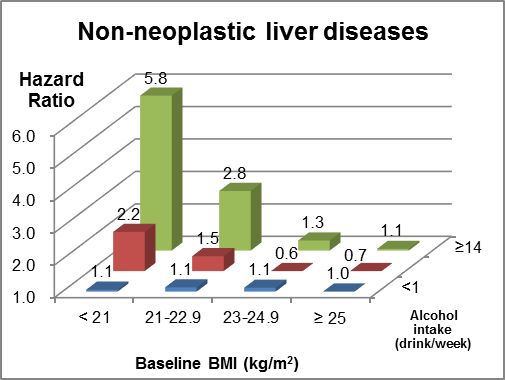

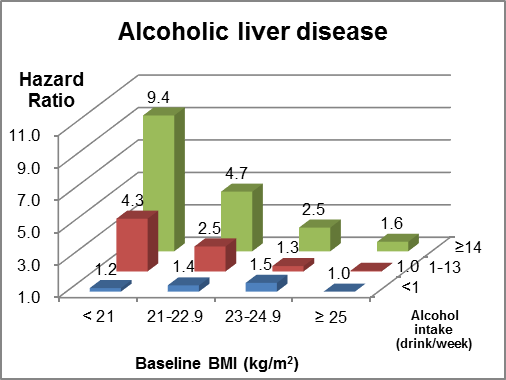

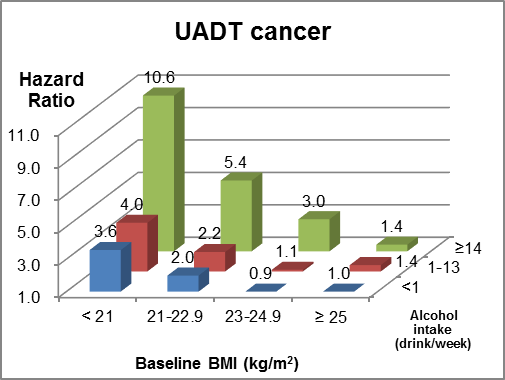

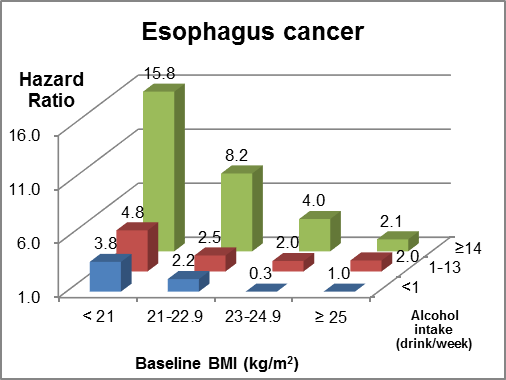

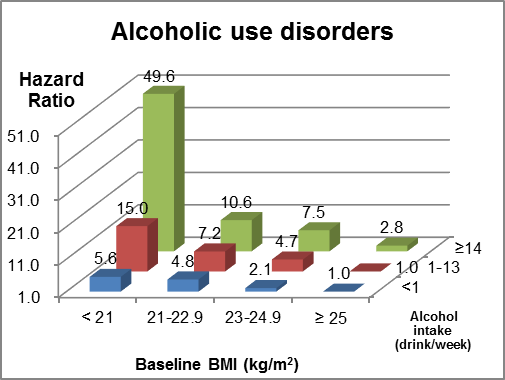


**eFigure 4. Hazard ratios of cause-specific mortality according to 12 groups combining body mass index (BMI) and weekly alcohol consumption.** Hazard ratios were calculated using Cox proportional hazard models after adjusting for age, cigarette smoking, physical activity, and household income. Persons with a BMI ≥ 25 kg/m^2^ and weekly alcohol consumption of less than one drink were the reference group. One drink was defined as containing approximately 9 g of ethanol. For alcohol use disorders, persons with ≥ 25 kg/m^2^ and weekly alcohol consumption of 0–13 drinks (combining the fewer than one drink/week and 1–13 drinks/week groups) were the reference group, due to the absence of deaths in persons with a BMI ≥ 25 kg/m ^2^ who consumed fewer than one drink per week. UADT, upper aerodigestive tract.

0251658240


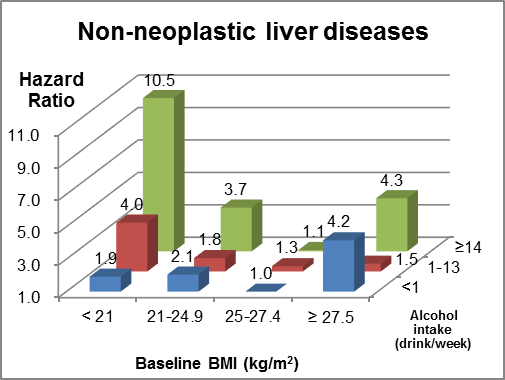

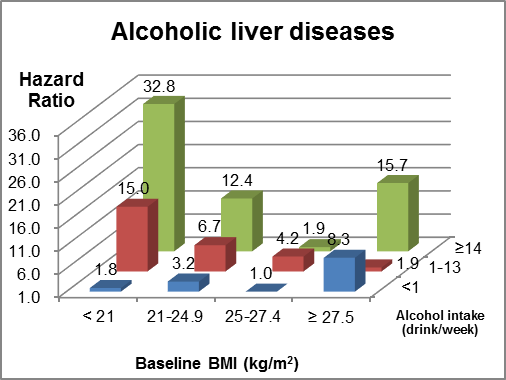

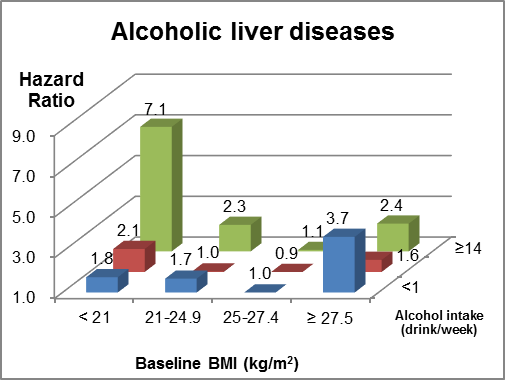

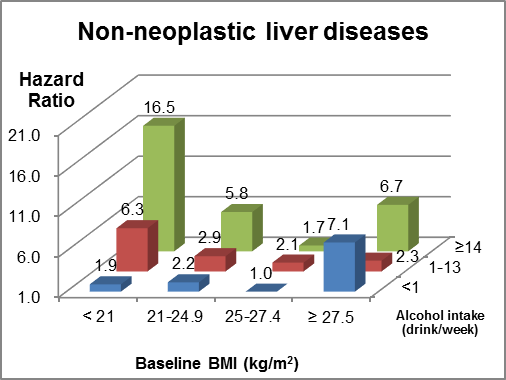

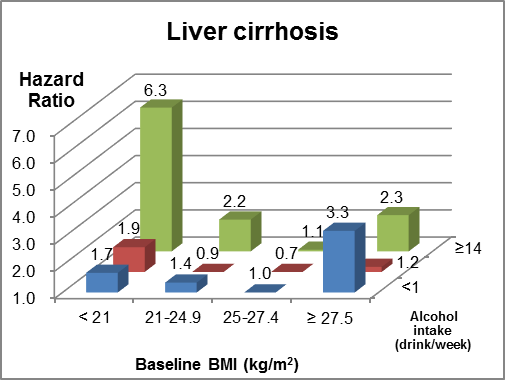

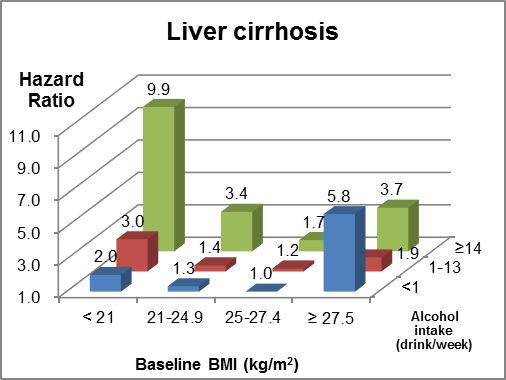


After excluding past drinkers

After excluding past drinkers

After excluding past drinkers

**eFigure 5. Hazard ratios of cause-specific mortality according to 12 groups combining body mass index (BMI) and weekly alcohol consumption in all participants and when past drinkers were excluded.** Hazard ratios were calculated using Cox proportional hazard models after adjusting for age, cigarette smoking, physical activity, and household income. Persons with a BMI of 25–27.4 kg/m^2^ and weekly alcohol consumption of less than one drink were the reference group. One drink was defined as containing approximately 9 g of ethanol.
