# Supplementary material for: Impact of alcohol consumption and body mass index on mortality from nonneoplastic liver diseases, upper aerodigestive tract cancers, and alcohol use disorders in Korean older middle-aged men: Prospective cohort study
Source: Medicine (Baltimore). 2016 Sep 30;95(39):e4876. doi: 10.1097/MD.0000000000004876 (PMC5265912; doi:10.1097/MD.0000000000004876)
Supplement: Supplemental Digital Content [file medi-95-e4876-s002.doc]

eTable 1. Death rates and adjusted^a^ hazard ratios for cause-specific mortality by weekly alcohol consumption among Korean older middle-aged men after excluding past drinkers.

| Cause of death (ICD-10) | Alcohol consumption per week | No. of deaths | Death rate^b^ | P-value | HR | (95% CI) |
| --- | --- | --- | --- | --- | --- | --- |
| Non-neoplastic liver | <1 drink | 46 | 274 |  | 1.00 | (Reference) |
| diseases (K70–K76) | 1–6 drinks | 60 | 306 | 0.217 | 1.28 | (0.87–1.88) |
|  | 7–13 drinks | 50 | 527 | <0.001 | 2.17 | (1.45–3.25) |
|  | 14–27 drinks | 68 | 691 | <0.001 | 2.73 | (1.86–3.98) |
|  | ≥28 drinks | 79 | 1156 | <0.001 | 4.12 | (2.84–5.97) |
|  | 5-drink increase per week | 303 | 484 | <0.001 | 1.69 | (1.52–1.88) |
| Alcoholic liver disease | <1 drink | 15 | 89 |  | 1.00 | (Reference) |
| (K70) | 1–6 drinks | 33 | 168 | 0.014 | 2.15 | (1.17–3.97) |
|  | 7–13 drinks | 31 | 327 | <0.001 | 3.99 | (2.15–7.43) |
|  | 14–27 drinks | 39 | 396 | <0.001 | 4.52 | (2.48–8.27) |
|  | ≥28 drinks | 45 | 659 | <0.001 | 6.53 | (3.61–11.82) |
|  | 5-drink increase per week | 163 | 261 | <0.001 | 1.70 | (1.48–1.96) |
| Upper aerodigestive tract | <1 drink | 27 | 161 |  | 1.00 | (Reference) |
| cancer (C00–C15, C30–C32) | 1–6 drinks | 41 | 209 | 0.157 | 1.42 | (0.87–2.32) |
|  | 7–13 drinks | 25 | 264 | 0.038 | 1.79 | (1.03–3.10) |
|  | 14–27 drinks | 46 | 467 | <0.001 | 3.11 | (1.92–5.04) |
|  | ≥28 drinks | 42 | 615 | <0.001 | 3.92 | (2.40–6.43) |
|  | 5-drink increase per week | 181 | 289 | <0.001 | 1.65 | (1.42–1.93) |
| Esophagus cancer (C15) | <1 drink | 12 | 71 |  | 1.00 | (Reference) |
|  | 1–6 drinks | 21 | 107 | 0.181 | 1.63 | (0.80–3.31) |
|  | 7–13 drinks | 16 | 169 | 0.014 | 2.57 | (1.21–5.46) |
|  | 14–27 drinks | 26 | 264 | <0.001 | 4.01 | (2.01–8.03) |
|  | ≥28 drinks | 27 | 395 | <0.001 | 5.89 | (2.95–11.78) |
|  | 5-drink increase per week | 102 | 163 | <0.001 | 1.81 | (1.52–2.14) |
| Alcohol use disorder | <1 drink | 5 | 30 |  | 1.00 | (Reference) |
| (F10) | 1–6 drinks | 9 | 46 | 0.262 | 1.88 | (0.62–5.64) |
|  | 7–13 drinks | 5 | 53 | 0.250 | 2.08 | (0.60–7.28) |
|  | 14–27 drinks | 9 | 91 | 0.030 | 3.42 | (1.12–10.38) |
|  | ≥28 drinks | 13 | 190 | 0.001 | 5.95 | (2.07–17.1) |
|  | 5-drink increase per week | 41 | 66 | 0.001 | 1.65 | (1.23–2.23) |

CI, confidence interval; HR, hazard ratio; ICD-10, International Classification of Diseases, 10th revision

a Adjusted for age at entry, smoking status, physical activity, household income, and body mass index.

b Crude death rate per 1,000,000 person-years.

eTable 2. Death rates and adjusted^a^ hazard ratios for cause-specific mortality by weekly alcohol consumption among Korean older middle-aged men without prevalent diseases relevant to the outcomes.

| Cause of death (ICD-10) | Alcohol consumption per week | No. of deaths | Death rate^b^ | P-value | HR | (95% CI) | Excluded diseases at baseline (no. of men after exclusion) |
| --- | --- | --- | --- | --- | --- | --- | --- |
| Non-neoplastic liver | <1 drink | 14 | 93 |  | 1.00 | (Reference) | Liver disease^c^ (K70–K76) |
| diseases (K70–K76) | 1–6 drinks | 15 | 108 | 0.417 | 1.35 | (0.65–2.82) | (n=75,506) |
|  | 7–13 drinks | 11 | 166 | 0.080 | 2.04 | (0.92–4.52) |  |
|  | 14–27 drinks | 23 | 336 | <0.001 | 3.83 | (1.95–7.53) |  |
|  | ≥28 drinks | 25 | 538 | <0.001 | 5.53 | (2.83–10.8) |  |
|  | 5-drink increase per week | 88 | 187 | <0.001 | 1.77 | (1.46–2.14) |  |
| Alcoholic liver disease | <1 drink | 3 | 20 |  | 1.00 | (Reference) | Liver disease^c^ (K70–K76) |
| (K70) | 1–6 drinks | 8 | 57 | 0.061 | 3.58 | (0.94–13.59) | (n=75,506) |
|  | 7–13 drinks | 6 | 91 | 0.018 | 5.41 | (1.34–21.89) |  |
|  | 14–27 drinks | 9 | 131 | 0.004 | 6.89 | (1.84–25.8) |  |
|  | ≥28 drinks | 16 | 344 | <0.001 | 15.35 | (4.39–53.63) |  |
|  | 5-drink increase per week | 42 | 89 | <0.001 | 1.81 | (1.42–2.32) |  |
| Upper aerodigestive tract | <1 drink | 30 | 142 |  | 1.00 | (Reference) | Upper aerodigestive tract |
| cancer (C00–C15, C30–C32) | 1–6 drinks | 32 | 163 | 0.411 | 1.23 | (0.75–2.04) | cancer^d^ (n=107,508) |
|  | 7–13 drinks | 22 | 232 | 0.055 | 1.72 | (0.99–3.01) |  |
|  | 14–27 drinks | 42 | 427 | <0.001 | 3.11 | (1.93–5.01) |  |
|  | ≥28 drinks | 37 | 542 | <0.001 | 3.81 | (2.33–6.24) |  |
|  | 5-drink increase per week | 163 | 244 | <0.001 | 1.70 | (1.44–1.99) |  |
| Esophagus cancer (C15) | <1 drink | 15 | 71 |  | 1.00 | (Reference) | Esophagus cancer^d^ |
|  | 1–6 drinks | 19 | 97 | 0.267 | 1.47 | (0.74–2.9) | (n=107,700) |
|  | 7–13 drinks | 15 | 158 | 0.018 | 2.38 | (1.16–4.91) |  |
|  | 14–27 drinks | 26 | 264 | <0.001 | 3.97 | (2.08–7.58) |  |
|  | ≥28 drinks | 25 | 366 | <0.001 | 5.42 | (2.82–10.44) |  |
|  | 5-drink increase per week | 100 | 149 | <0.001 | 1.84 | (1.55–2.18) |  |
| Alcohol use disorder | <1 drink | 3 | 14 |  | 1.00 | (Reference) | Substance use disorder^c^ |
| (F10) | 1–6 drinks | 6 | 31 | 0.175 | 2.63 | (0.65–10.64) | (F10–F19; n=106,126) |
|  | 7–13 drinks | 4 | 43 | 0.106 | 3.48 | (0.77–15.83) |  |
|  | 14–27 drinks | 6 | 62 | 0.030 | 4.78 | (1.16–19.64) |  |
|  | ≥28 drinks | 8 | 121 | 0.002 | 8.20 | (2.10–31.99) |  |
|  | 5-drink increase per week | 27 | 41 | 0.006 | 1.72 | (1.17–2.54) |  |

CI, confidence interval; HR, hazard ratio; ICD–10, International Classification of Diseases, 10th revision

a Adjusted for age at entry, smoking status, physical activity, household income, and body mass index.

b Crude death rate per 1,000,000 person-years.

c Participants were considered to have prevalent diseases, which were identified by the primary diagnosis and the first comorbidity in the National Health Insurance claims data, if they visited a medical institution at least once between January 1, 2000 and July 31, 2004.

d These cancers were diagnosed from January 1, 1992 to July 31, 2004 and were assessed using the National Cancer Incidence Database.

eTable 3. Death rates and adjusted^a^ hazard ratios for cause-specific mortality by alcohol consumption frequency among the Korean older middle-aged men.

| Cause of death (ICD-10) | Alcohol consumption frequency | No. of deaths | Death rate^b^ | P-value | HR | (95% CI) |
| --- | --- | --- | --- | --- | --- | --- |
| Non-neoplastic liver | 1 or less/month | 79 | 379 |  | 1.00 | (Reference) |
| diseases (K70–K76) | 2-3 day/month | 32 | 254 | 0.212 | 0.77 | (0.51–1.16) |
|  | 1-2 day/week | 51 | 349 | 0.654 | 1.08 | (0.76–1.55) |
|  | 3-4 day/week | 66 | 570 | 0.007 | 1.58 | (1.13–2.20) |
|  | 5-7 day/week | 110 | 1493 | <0.001 | 3.47 | (2.58–4.67) |
|  | One drinking day increase per week | 338 | 505 | <0.001 | 1.24 | (1.19–1.29) |
| Alcoholic liver disease | 1 or less/month | 30 | 144 |  | 1.00 | (Reference) |
| (K70) | 2-3 day/month | 14 | 111 | 0.712 | 0.89 | (0.47–1.68) |
|  | 1-2 day/week | 32 | 219 | 0.027 | 1.77 | (1.07–2.92) |
|  | 3-4 day/week | 40 | 345 | <0.001 | 2.38 | (1.47–3.85) |
|  | 5-7 day/week | 62 | 842 | <0.001 | 4.62 | (2.96–7.21) |
|  | One drinking day increase per week | 178 | 266 | <0.001 | 1.28 | (1.21–1.36) |
| Upper aerodigestive tract | 1 or less/month | 53 | 255 |  | 1.00 | (Reference) |
| cancer (C00–C15, C30–C32) | 2-3 day/month | 15 | 119 | 0.027 | 0.52 | (0.29-0.93) |
|  | 1-2 day/week | 35 | 240 | 0.700 | 1.09 | (0.71–1.68) |
|  | 3-4 day/week | 42 | 363 | 0.046 | 1.52 | (1.01–2.29) |
|  | 5-7 day/week | 54 | 733 | <0.001 | 2.64 | (1.79–3.89) |
|  | One drinking day increase per week | 199 | 297 | <0.001 | 1.19 | (1.13–1.26) |
| Esophagus cancer (C15) | 1 or less/month | 23 | 110 |  | 1.00 | (Reference) |
|  | 2-3 day/month | 8 | 63 | 0.274 | 0.64 | (0.28–1.43) |
|  | 1-2 day/week | 17 | 116 | 0.548 | 1.21 | (0.64–2.29) |
|  | 3-4 day/week | 25 | 216 | 0.012 | 2.09 | (1.18–3.71) |
|  | 5-7 day/week | 35 | 475 | <0.001 | 4.05 | (2.37–6.93) |
|  | One drinking day increase per week | 108 | 161 | <0.001 | 1.27 | (1.18–1.37) |
| Alcohol use disorder | 1 or less/month | 5 | 24 |  | 1.00 | (Reference) |
| (F10) | 2-3 day/month | 5 | 40 | 0.250 | 2.08 | (0.60–7.23) |
|  | 1-2 day/week | 7 | 48 | 0.111 | 2.57 | (0.81–8.20) |
|  | 3-4 day/week | 13 | 112 | 0.003 | 5.03 | (1.76–14.3) |
|  | 5-7 day/week | 11 | 149 | 0.004 | 4.94 | (1.68–14.5) |
|  | per a day increase/week | 41 | 61 | 0.002 | 1.21 | (1.07–1.37) |

CI, confidence interval; HR, hazard ratio; ICD–10, International Classification of Diseases, 10th revision.

a Adjusted for age at entry, smoking status, physical activity, household income, and body mass index.

b Crude death rate per 1,000,000 person-years.

eTable 4. Death rates and adjusted^a^ hazard ratios for cause-specific mortality by alcohol consumption per drinking day among Korean older middle-aged men.

| Cause of death (ICD-10) | Alcohol consumption per drinking day | No. of deaths | Death rate^b^ | P-value | HR | (95% CI) |
| --- | --- | --- | --- | --- | --- | --- |
| Non-neoplastic liver | <2 drinks | 75 | 459 |  | 1.00 | (Reference) |
| diseases (K70–K76) | 2–3 drinks | 87 | 408 | 0.647 | 0.93 | (0.68–1.27) |
|  | 4–5 drinks | 49 | 438 | 0.682 | 1.08 | (0.75–1.56) |
|  | 6–7 drinks | 83 | 734 | 0.001 | 1.75 | (1.27–2.41) |
|  | ≥8 drinks | 44 | 644 | 0.019 | 1.58 | (1.08–2.32) |
|  | 5-drink increase per drinking day | 338 | 505 | <0.001 | 1.25 | (1.11–1.4) |
| Alcoholic liver disease | <2 drinks | 31 | 190 |  | 1.00 | (Reference) |
| (K70) | 2–3 drinks | 48 | 225 | 0.423 | 1.20 | (0.76–1.9) |
|  | 4–5 drinks | 24 | 215 | 0.485 | 1.21 | (0.71–2.08) |
|  | 6–7 drinks | 47 | 416 | 0.001 | 2.22 | (1.4–3.54) |
|  | ≥8 drinks | 28 | 410 | 0.002 | 2.25 | (1.33–3.8) |
|  | 5-drink increase per drinking day | 178 | 266 | <0.001 | 1.31 | (1.13–1.52) |
| Upper aerodigestive tract | <2 drinks | 30 | 183 |  | 1.00 | (Reference) |
| cancer (C00–C15, C30–C32) | 2–3 drinks | 63 | 296 | 0.018 | 1.70 | (1.09–2.63) |
|  | 4–5 drinks | 32 | 286 | 0.019 | 1.83 | (1.1–3.04) |
|  | 6–7 drinks | 44 | 389 | <0.001 | 2.55 | (1.59–4.11) |
|  | ≥8 drinks | 30 | 439 | <0.001 | 3.10 | (1.84–5.22) |
|  | 5-drink increase per drinking day | 199 | 297 | <0.001 | 1.34 | (1.17–1.54) |
| Esophagus cancer (C15) | <2 drinks | 12 | 73 |  | 1.00 | (Reference) |
|  | 2–3 drinks | 30 | 141 | 0.041 | 2.02 | (1.03–3.96) |
|  | 4–5 drinks | 20 | 179 | 0.004 | 2.90 | (1.4–5.99) |
|  | 6–7 drinks | 28 | 248 | <0.001 | 4.16 | (2.08–8.32) |
|  | ≥8 drinks | 18 | 264 | <0.001 | 4.81 | (2.28– 10.17) |
|  | 5-drink increase per drinking day | 108 | 161 | <0.001 | 1.46 | (1.26–1.69) |
| Alcohol use disorder | <2 drinks | 3 | 18 |  | 1.00 | (Reference) |
| (F10) | 2–3 drinks | 9 | 42 | 0.156 | 2.59 | (0.7–9.65) |
|  | 4–5 drinks | 8 | 72 | 0.021 | 4.90 | (1.28– 18.82) |
|  | 6–7 drinks | 10 | 88 | 0.007 | 6.06 | (1.62–22.57) |
|  | ≥8 drinks | 11 | 161 | <0.001 | 10.99 | (2.98–40.51) |
|  | 5-drink increase per drinking day | 41 | 61 | <0.001 | 1.72 | (1.48–1.99) |

CI, confidence interval; HR, hazard ratio; ICD-10, International Classification of Diseases, 10th revision.

a Adjusted for age at entry, smoking status, physical activity, household income, and body mass index.

b Crude death rate per 1,000,000 person-years.

eTable 5. Death rates and adjusted^a^ hazard ratios for cause-specific mortality by body mass index (BMI) among Korean older middle-aged men (corresponding to Figure 1).

| Cause of death (ICD-10) | BMI (kg/m^2^) | No. of deaths | Death rate^b^ | P-value | HR | (95% CI) |
| --- | --- | --- | --- | --- | --- | --- |
| Non-neoplastic liver | 12.0–18.4 | 32 | 2024 | <0.001 | 7.01 | (4.20–11.69) |
| diseases (K70-K76) | 18.5–20.9 | 84 | 987 | <0.001 | 3.78 | (2.47–5.80) |
|  | 21.0–22.9 | 101 | 623 | <0.001 | 2.72 | (1.80–4.13) |
|  | 23.0–24.9 | 65 | 309 | 0.064 | 1.51 | (0.98–2.34) |
|  | 25.0–27.4 | 29 | 196 |  | 1.00 | (Reference) |
|  | 27.5–29.9 | 19 | 471 | 0.005 | 2.28 | (1.28–4.07) |
|  | 30.0–47 | 8 | 969 | <0.001 | 4.39 | (2.01–9.61) |
|  | 5 kg/m^2^ higher BMI | 338 | 505 | <0.001 | 0.46 | (0.37-0.56) |
| Alcoholic liver disease | 12.0–18.4 | 17 | 1075 | <0.001 | 8.67 | (4.02–18.72) |
| (K70) | 18.5–20.9 | 50 | 587 | <0.001 | 5.31 | (2.75–10.26) |
|  | 21.0–22.9 | 53 | 327 | <0.001 | 3.50 | (1.82–6.71) |
|  | 23.0–24.9 | 36 | 171 | 0.026 | 2.15 | (1.09–4.23) |
|  | 25.0–27.4 | 11 | 74 |  | 1.00 | (Reference) |
|  | 27.5–29.9 | 9 | 223 | 0.019 | 2.86 | (1.18–6.90) |
|  | 30.0–47 | 2 | 242 | 0.173 | 2.85 | (0.63–12.87) |
|  | 5 kg/m^2^ higher BMI | 178 | 266 | <0.001 | 0.40 | (0.30-0.53) |
| Upper aerodigestive tract | 12.0–18.4 | 18 | 1138 | <0.001 | 6.33 | (3.33–12.05) |
| cancer (C00-C15, C30-C32) | 18.5–20.9 | 57 | 670 | <0.001 | 3.99 | (2.40–6.64) |
|  | 21.0–22.9 | 59 | 364 | 0.001 | 2.32 | (1.41–3.83) |
|  | 23.0–24.9 | 37 | 176 | 0.507 | 1.20 | (0.70–2.05) |
|  | 25.0–27.4 | 21 | 142 |  | 1.00 | (Reference) |
|  | 27.5–29.9 | 6 | 149 | 0.957 | 1.03 | (0.41–2.54) |
|  | 30.0–47 | 1 | 121 | 0.849 | 0.82 | (0.11–6.12) |
|  | 5 kg/m^2^ higher BMI | 199 | 297 | <0.001 | 0.32 | (0.24-0.42) |
| Esophagus cancer (C15) | 12.0–18.4 | 5 | 316 | 0.041 | 2.98 | (1.04–8.50) |
|  | 18.5–20.9 | 35 | 411 | <0.001 | 4.06 | (2.12–7.77) |
|  | 21.0–22.9 | 32 | 197 | 0.029 | 2.06 | (1.08–3.94) |
|  | 23.0–24.9 | 20 | 95 | 0.884 | 1.05 | (0.52–2.12) |
|  | 25.0–27.4 | 13 | 88 |  | 1.00 | (Reference) |
|  | 27.5–29.9 | 3 | 74 | 0.760 | 0.82 | (0.23–2.89) |
|  | 30.0–47 | 0 | 0 |  | 0.00 |  |
|  | 5 kg/m^2^ higher BMI | 108 | 161 | <0.001 | 0.36 | (0.25-0.52) |
| Alcohol use disorder | 12.0–18.4 | 10 | 632 | <0.001 | 58.39 | (7.32–465.6) |
| (F10) | 18.5–20.9 | 12 | 141 | 0.011 | 14.50 | (1.87–112.6) |
|  | 21.0–22.9 | 10 | 62 | 0.056 | 7.47 | (0.95–58.58) |
|  | 23.0–24.9 | 7 | 33 | 0.148 | 4.70 | (0.58–38.26) |
|  | 25.0–27.4 | 1 | 7 |  | 1.00 | (Reference) |
|  | 27.5–29.9 | 1 | 25 | 0.375 | 3.51 | (0.22–56.15) |
|  | 30.0–47 | 0 | 0 |  | 0.00 |  |
|  | 5 kg/m^2^ higher BMI | 41 | 61 | <0.001 | 0.18 | (0.10-0.33) |

CI, confidence interval; HR, hazard ratio; ICD-10, International Classification of Diseases, 10th revision.

a Adjusted for age at entry, smoking status, physical activity, household income, and body mass index.

b Crude death rate per 1,000,000 person-years.

eTable 6. Death rates and adjusted^a^ hazard ratios for cause-specific mortality by body mass index (BMI) among older middle-aged men without prevalent diseases relevant to the outcomes.

| Cause of death (ICD–10) | BMI (kg/m^2^) | No. of deaths | Death rate^b^ | P-value | HR | (95% CI) | Excluded diseases at baseline (no. of men after exclusion) |
| --- | --- | --- | --- | --- | --- | --- | --- |
| Non-neoplastic liver | 12.0–18.4 | 9 | 797 | <0.001 | 5.69 | (2.22–14.6) | Liver disease^c^ (K70–K76) |
| diseases (K70-K76) | 18.5–20.9 | 28 | 452 | 0.001 | 3.53 | (1.65–7.55) | (n=75,506) |
|  | 21.0–22.9 | 26 | 222 | 0.073 | 2.01 | (0.94–4.30) |  |
|  | 23.0–24.9 | 12 | 80 | 0.681 | 0.83 | (0.35–1.98) |  |
|  | 25.0–27.4 | 9 | 90 |  | 1.00 | (Reference) |  |
|  | 27.5–29.9 | 3 | 114 | 0.801 | 1.18 | (0.32–4.37) |  |
|  | 30.0–47 | 1 | 194 | 0.563 | 1.84 | (0.23–14.54) |  |
|  | 5 kg/m^2^ higher BMI | 88 | 187 | <0.001 | 0.37 | (0.25-0.56) |  |
| Alcoholic liver disease | 12.0–18.4 | 6 | 531 | 0.002 | 9.62 | (2.35–39.41) | Liver disease^c^ (K70–K76) |
| (K70) | 18.5–20.9 | 14 | 226 | 0.017 | 4.62 | (1.31–16.26) | (n=75,506) |
|  | 21.0–22.9 | 13 | 111 | 0.117 | 2.74 | (0.78–9.68) |  |
|  | 23.0–24.9 | 5 | 33 | 0.995 | 1.00 | (0.24–4.21) |  |
|  | 25.0–27.4 | 3 | 30 |  | 1.00 | (Reference) |  |
|  | 27.5–29.9 | 1 | 38 | 0.902 | 1.15 | (0.12–11.1) |  |
|  | 30.0–47 | 0 | 0 |  | 0.00 |  |  |
|  | 5 kg/m^2^ higher BMI | 42 | 89 | <0.001 | 0.28 | (0.15-0.50) |  |
| Upper aerodigestive tract | 12.0–18.4 | 11 | 700 | <0.001 | 4.14 | (1.94–8.83) | Upper aerodigestive tract |
| cancer (C00-C15, C30-C32) | 18.5–20.9 | 44 | 518 | <0.001 | 3.27 | (1.89–5.65) | cancer^d^ (n=107,508) |
|  | 21.0–22.9 | 49 | 303 | 0.008 | 2.06 | (1.21–3.51) |  |
|  | 23.0–24.9 | 33 | 157 | 0.602 | 1.16 | (0.66–2.04) |  |
|  | 25.0–27.4 | 19 | 129 |  | 1.00 | (Reference) |  |
|  | 27.5–29.9 | 6 | 149 | 0.777 | 1.14 | (0.46–2.86) |  |
|  | 30.0–47 | 1 | 121 | 0.939 | 0.92 | (0.12–6.91) |  |
|  | 5 kg/m^2^ higher BMI | 163 | 244 | <0.001 | 0.41 | (0.30-0.55) |  |
| Esophagus cancer (C15) | 12.0–18.4 | 4 | 253 | 0.146 | 2.33 | (0.75–7.26) | Esophagus cancer^d^ |
|  | 18.5–20.9 | 31 | 364 | <0.001 | 3.51 | (1.82–6.8) | (n=107,700) |
|  | 21.0–22.9 | 29 | 179 | 0.073 | 1.83 | (0.94–3.53) |  |
|  | 23.0–24.9 | 20 | 95 | 0.908 | 1.04 | (0.52–2.10) |  |
|  | 25.0–27.4 | 13 | 88 |  | 1.00 | (Reference) |  |
|  | 27.5–29.9 | 3 | 74 | 0.768 | 0.83 | (0.24–2.91) |  |
|  | 30.0–47 | 0 | 0 | 0.985 | 0.00 |  |  |
|  | 5 kg/m^2^ higher BMI | 100 | 149 | <0.001 | 0.41 | (0.27-0.60) |  |
| Alcohol use disorder | 12.0–18.4 | 6 | 395 | 0.001 | 39.05 | (4.57–334.1) | Substance use disorder^c^ |
| (F10) | 18.5–20.9 | 9 | 108 | 0.022 | 11.34 | (1.42–90.83) | (F10–F19; n=106,126) |
|  | 21.0–22.9 | 7 | 44 | 0.120 | 5.29 | (0.65–43.25) |  |
|  | 23.0–24.9 | 4 | 19 | 0.378 | 2.68 | (0.30–24.0) |  |
|  | 25.0–27.4 | 1 | 7 |  | 1.00 | (Reference) |  |
|  | 27.5–29.9 | 0 | 0 |  | 0.00 |  |  |
|  | 30.0–47 | 0 | 0 |  | 0.00 |  |  |
|  | 5 kg/m^2^ higher BMI | 27 | 41 | <0.001 | 0.15 | (0.07–0.32) |  |

CI, confidence interval; HR, hazard ratio; ICD-10, International Classification of Diseases, 10th revision

a Adjusted for age at entry, smoking status, physical activity, household income, and body mass index.

b Crude death rate per 1,000,000 person-years.

c Participants were considered to have prevalent diseases if those diseases were identified as the primary diagnosis or the first comorbidity in the National Health Insurance claims data for at least one visit to a medical institution between January 1, 2000 and July 31, 2004.

d These cancers were diagnosed from January 1, 1992 to July 31, 2004 and were assessed through the National Cancer Incidence Database.

eTable 7. Death rates and adjusted^a^ hazard ratios for cause-specific mortality by weekly alcohol consumption according to BMI among Korean older middle-aged men.

|  |  | **All participants (12–47 kg/m^2^)** | | | | | **12–20.9 kg/m^2^** | | | | | **21–24.9 kg/m^2^** | | | | | **25–47 kg/m^2^** | | | | |
| --- | --- | --- | --- | --- | --- | --- | --- | --- | --- | --- | --- | --- | --- | --- | --- | --- | --- | --- | --- | --- | --- |
| **Causes of death** | **Alcohol consumption per week** | **No. of deaths** | **Death rate^b^** | **P-**  **value** | **HR (95% CI)** | | **No. of deaths** | **Death rate^b^** | **P-**  **value** | **HR (95% CI)** | | **No. of deaths** | **Death rate^b^** | **P-**  **value** | **HR (95% CI)** | | **No. of deaths** | **Death rate^b^** | **P-**  **value** | **HR (95% CI)** | |
| Non-neoplastic | <1 drink | 81 | 382 |  | 1.00 | (Reference) | 16 | 443 |  | 1.00 | (Reference) | 45 | 389 |  | 1.00 | (Reference) | 20 | 331 |  | 1.00 | (Reference) |
| liver diseases | 1–13 drinks | 110 | 378 | 0.556 | 1.09 | (0.82–1.46) | 37 | 902 | 0.009 | 2.20 | (1.22–3.99) | 54 | 327 | 0.499 | 0.87 | (0.58–1.3) | 19 | 224 | 0.481 | 0.80 | (0.42–1.5) |
| (K70–K76) | ≥14 drinks | 147 | 882 | <0.001 | 2.32 | (1.76–3.07) | 63 | 2645 | <0.001 | 5.92 | (3.36–10.4) | 67 | 731 | 0.005 | 1.74 | (1.18–2.57) | 17 | 332 | 0.767 | 1.11 | (0.57–2.15) |
|  | One-category increase | 338 | 505 | <0.001 | 1.59 | (1.37–1.84) | 116 | 1149 | <0.001 | 2.50 | (1.91–3.26) | 166 | 446 | 0.003 | 1.37 | (1.11–1.68) | 56 | 285 | 0.811 | 1.04 | (0.74–1.48) |
|  | 5-drink increase | 338 | 505 | <0.001 | 1.70 | (1.52–1.89) | 116 | 1149 | <0.001 | 1.76 | (1.5–2.07) | 166 | 446 | <0.001 | 1.70 | (1.44–2.02) | 56 | 285 | 0.033 | 1.41 | (1.03–1.95) |
| Alcoholic liver | <1 drink | 30 | 141 |  | 1.00 | (Reference) | 6 | 166 |  | 1.00 | (Reference) | 18 | 156 |  | 1.00 | (Reference) | 6 | 99 |  | 1.00 | (Reference) |
| disease | 1–13 drinks | 64 | 220 | 0.019 | 1.69 | (1.09–2.62) | 24 | 585 | 0.005 | 3.60 | (1.46–8.85) | 32 | 194 | 0.372 | 1.30 | (0.73–2.34) | 8 | 94 | 0.917 | 1.06 | (0.36–3.09) |
| (K70) | ≥14 drinks | 84 | 504 | <0.001 | 3.30 | (2.16–5.06) | 37 | 1554 | <0.001 | 8.30 | (3.45–20.0) | 39 | 425 | 0.003 | 2.40 | (1.35–4.27) | 8 | 156 | 0.456 | 1.51 | (0.51–4.46) |
|  | One-category increase | 178 | 266 | <0.001 | 1.84 | (1.5–2.27) | 67 | 664 | <0.001 | 2.66 | (1.86–3.82) | 89 | 239 | 0.002 | 1.59 | (1.19–2.13) | 22 | 112 | 0.447 | 1.24 | (0.71–2.16) |
|  | 5-drink increase | 178 | 266 | <0.001 | 1.70 | (1.47–1.96) | 67 | 664 | <0.001 | 1.82 | (1.51–2.2) | 89 | 239 | <0.001 | 1.65 | (1.31–2.07) | 22 | 112 | 0.379 | 1.28 | (0.74–2.23) |
| UADT cancer | <1 drink | 45 | 212 |  | 1.00 | (Reference) | 18 | 498 |  | 1.00 | (Reference) | 20 | 173 |  | 1.00 | (Reference) | 7 | 116 |  | 1.00 | (Reference) |
| (C00–C15, | 1–13 drinks | 66 | 227 | 0.372 | 1.19 | (0.81–1.75) | 22 | 536 | 0.490 | 1.25 | (0.66–2.35) | 31 | 188 | 0.606 | 1.16 | (0.66–2.05) | 13 | 153 | 0.795 | 1.13 | (0.45–2.86) |
| C30–C32) | ≥14 drinks | 88 | 528 | <0.001 | 2.72 | (1.87–3.94) | 35 | 1470 | <0.001 | 3.52 | (1.94–6.39) | 45 | 491 | <0.001 | 2.95 | (1.71–5.08) | 8 | 156 | 0.923 | 1.05 | (0.38–2.95) |
|  | One-category increase | 199 | 297 | <0.001 | 1.72 | (1.42–2.09) | 75 | 743 | <0.001 | 1.97 | (1.44–2.7) | 96 | 258 | <0.001 | 1.83 | (1.38–2.44) | 28 | 142 | 0.932 | 1.02 | (0.62–1.69) |
|  | 5-drink increase | 199 | 297 | <0.001 | 1.64 | (1.4–1.93) | 75 | 743 | <0.001 | 1.63 | (1.29–2.06) | 96 | 258 | <0.001 | 1.83 | (1.45–2.29) | 28 | 142 | 0.961 | 1.02 | (0.49–2.12) |
| Esophagus | <1 drink | 18 | 85 |  | 1.00 | (Reference) | 8 | 222 |  | 1.00 | (Reference) | 7 | 61 |  | 1.00 | (Reference) | 3 | 50 |  | 1.00 | (Reference) |
| cancer | 1–13 drinks | 37 | 127 | 0.074 | 1.68 | (0.95–2.97) | 11 | 268 | 0.475 | 1.40 | (0.56–3.52) | 18 | 109 | 0.142 | 1.93 | (0.8–4.66) | 8 | 94 | 0.492 | 1.60 | (0.42–6.09) |
| (C15) | ≥14 drinks | 53 | 318 | <0.001 | 4.20 | (2.43–7.28) | 21 | 882 | <0.001 | 4.68 | (2–10.93) | 27 | 294 | <0.001 | 5.28 | (2.26– 12.3) | 5 | 98 | 0.579 | 1.51 | (0.35–6.42) |
|  | One-category increase | 108 | 161 | <0.001 | 2.14 | (1.63–2.81) | 40 | 396 | <0.001 | 2.34 | (1.51–3.63) | 52 | 140 | <0.001 | 2.41 | (1.61–3.62) | 16 | 81 | 0.611 | 1.19 | (0.61–2.33) |
|  | 5-drink increase | 108 | 161 | <0.001 | 1.83 | (1.54–2.17) | 40 | 396 | <0.001 | 1.80 | (1.39–2.33) | 52 | 140 | <0.001 | 2.03 | (1.57–2.63) | 16 | 81 | 0.475 | 1.30 | (0.63–2.71) |
| Alcohol use | <1 drink | 5 | 24 |  | 1.00 | (Reference) | 2 | 55 |  | 1.00 | (Reference) | 3 | 26 |  | 1.00 | (Reference) | 0 | 0 |  |  |  |
| disorder | 1–13 drinks | 14 | 48 | 0.097 | 2.39 | (0.85–6.71) | 6 | 146 | 0.186 | 2.99 | (0.59–15.1) | 7 | 42 | 0.403 | 1.79 | (0.46–7.01) | 1 | 12 |  |  |  |
| (F10) | ≥14 drinks | 22 | 132 | 0.001 | 5.58 | (2.06–15.1) | 14 | 588 | 0.004 | 9.48 | (2.06–43.7) | 7 | 76 | 0.119 | 3.01 | (0.75–12.0) | 1 | 20 |  |  |  |
|  | One-category increase | 41 | 61 | <0.001 | 2.35 | (1.5–3.69) | 22 | 218 | 0.001 | 3.11 | (1.6–6.04) | 17 | 46 | 0.107 | 1.72 | (0.89–3.35) | 2 | 10 | 0.542 | 1.96 | (0.22–17.2) |
|  | 5-drink increase | 41 | 61 | <0.001 | 1.71 | (1.28–2.29) | 22 | 218 | 0.002 | 1.77 | (1.24–2.52) | 17 | 46 | 0.217 | 1.50 | (0.79–2.85) | 2 | 10 | 0.278 | 1.57 | (0.69–3.55) |

BMI, body mass index; CI, confidence interval; HR, hazard ratio; ICD-10, International Classification of Diseases, 10th revision; UADT, upper aerodigestive tract.

a Adjusted for age at entry, smoking status, physical activity, household income, and body mass index.

b Crude death rate per 1,000,000 person-years
